# The Multifarious Role of Microglia in Brain Metastasis

**DOI:** 10.3389/fncel.2018.00414

**Published:** 2018-11-12

**Authors:** Manuel Sarmiento Soto, Nicola R. Sibson

**Affiliations:** ^1^Cancer Research UK and Medical Research Council Oxford Institute for Radiation Oncology, Department of Oncology, University of Oxford, Oxford, United Kingdom; ^2^Department of Biochemistry and Molecular Biology, University of Seville, Seville, Spain; ^3^John Fulcher Neuro-Oncology Laboratory, Department of Medicine, Imperial College London, London, United Kingdom

**Keywords:** microglia, macrophages, brain, metastasis, cancer, therapies

## Abstract

The immune landscape in brain metastasis is a very heterogeneous framework. Amongst a broad plethora of cells within the tumor microenvironment, the presence of activated microglia has been perfectly described. The innate role of microglial cells is to detect and eliminate any insults that may disturb the regular behavior of the brain. As part of its defensive role, it releases pro- and anti-inflammatory cytokines that aim to modulate the inflammatory scenario at the metastatic *foci*. However, the long term effects that these cells may exert on the metastatic progression is not clear. One of the biggest challenges in the field is to distinguish between brain resident microglial cells and infiltrated bone-marrow derived macrophages. Part of this issue is the fact that both cell types share similar phenotypes. Current studies are based on the modulation of the immune response against cancer cells (immunotherapy). However, most of current clinical trials and newly developed drugs focus on the adaptive immune response (e.g., immune blockade check-points). Additionally, the unique structure of the central nervous system with the presence of the blood-brain barrier have hindered a significant advance in novel therapies against brain metastasis. In this manuscript, we describe current advances in characterization of tumor-associated microglia and macrophages, the importance of microglia during the anti-cancerous response, and the future direction for the development of new strategies against this complex disease.

## Introduction

At the beginning of the last century, Río-Hortega (1919) was the first scientist to describe a new type of resident cells within the central nervous system (CNS), which he termed microglia. This was the first study that clearly differentiated these cells from the “third brain element" (glial cells) described by Ramón y Cajal (1913) years earlier. Even back then, owing to his interests in neuropathology, Hortega pioneered research into the important role of microglia in CNS tumors (Pérez-Cerdá et al., 2015).

Currently, the use of pre-clinical models has helped us to delve into the importance of this unique brain cell type. Microglia are mostly present in the gray matter, particularly in the hippocampus, olfactory telencephalon, basal ganglia, and substantia nigra. The distribution of these cells in adult mouse brains fluctuates from 5% in the cortex to 12% in the substantia nigra (Lawson et al., 1990; Yang et al., 2013). Nevertheless, the key role of microglia in the field of oncology is still an area of substantial investigation. Numerous primary cancers are able to colonize the brain. Specifically, lung, melanoma and breast cancer present the highest incidence of brain metastasis (Fidler, 2015). These patients have significantly increased life expectancy owing to better treatments for the primary tumor. However, this improvement comes with a higher risk of suffering from the colonization of distant organs. Therefore, rather than the primary origin, the metastatic spread of circulating tumor cells to distant sites (metastasis) is the major cause of cancer demise (Chaffer and Weinberg, 2011; Lambert et al., 2017). Consequently, the incidence of brain metastases (metastatic spread to the central nervous system, BM has become a significant clinical problem (Lowery and Yu, 2017). The poor patient life expectancy, measured just in months, because of the late diagnosis and the lack of efficient treatments, make this disease one of the biggest challenges in the 21st century.

However, it is worth mentioning that metastatic colonization is a very inefficient process in which most cells die and only a minimal population survive and successfully establish metastases (Weiss, 1990). The reasons for the poor efficacy of this multi-step process are shear stress within the blood vessels, immune surveillance and oxidative stress (Massagué and Obenauf, 2016), commonly led by microglial cells.

In this manuscript, we aim at describing the complex biology of microglial cells within the CNS during metastasis progression and the importance of microglia and macrophages as future components of anti-metastatic therapies.

## When the Privileged Immune Nature of the Brain Is Compromised

Despite recent findings concerning the role of the glymphatic system in the CNS (Louveau et al., 2017), the brain is still considered to possess a privileged immune environment owing to the presence of the blood-brain barrier (BBB) and its resident defensive system. The BBB impedes undesired factors in the blood and lymph from crossing into the brain parenchyma. Amongst the different components of the BBB, endothelial cells, pericytes, and astrocytes are the main players in CNS integrity (Persidsky et al., 2006). However, certain populations of circulating tumor cells have the ability to cross this ontogenetically evolved hurdle and thrive in the brain parenchyma.

It has long been described that tumor cells share the same abilities as leukocytes to cross into the brain parenchyma (Strell and Entschladen, 2008). During metastatic colonization, the structure of the BBB is transiently compromised by the paracellular and transcellular extravasation of tumor cells and the release of tissue-disrupting soluble factors (e.g., cytokines) (Wilhelm et al., 2013). After this transient disruption of the tight junction network, microglia can contribute to regulating BBB integrity (da Fonseca et al., 2014), and also repair the BBB following cerebrovascular damage in a purinergic receptor P2RY12-dependent manner (Lou et al., 2016). Microglia express high levels of P2RY12, which serve as chemotactic receptor at sites of CNS injury. Microglia can aggregate and form a physical barrier (via E-cadherin upregulation) that will engulf the damaged vessel wall and temporarily assume BBB functions.

Subsequently, during the development and growth phase of brain metastases, microglia show an impaired immune response evolving, it is thought, into a tumor-supportive phenotype (Pukrop et al., 2010; Roesch et al., 2018). Microglia and infiltrating macrophages are important components of the tumor microenvironment (TME). In regular homeostatic conditions, microglia remain resting or quiescent, but in response to disease or injury, including tumor cell invasion, they become activated. These cells can adopt many different states of polarization during cancer progression that fluctuates across a pro- to anti-inflammatory spectrum (Wang et al., 2014). Those immune cells with a pro-inflammatory phenotype have been described as exerting an anti-tumorigenic effect whilst those with an anti-inflammatory profile have shown tumor-supporting activity (Pukrop et al., 2010; Wang et al., 2014). The mechanisms underlying the phenotypic switch between these two states and the potential coexistence of both identities are far from being understood in a brain metastasis context. However, what it is clearly known is that, during systemic metastasis, cancer cells have the ability to drive surrounding immune cells into a tumor supportive phenotype (Sica et al., 2006; Hao et al., 2012).

## Microglia Versus Bone Marrow-Derived Macrophages (BMDM)

The activation of microglia surrounding metastatic *loci* has been clearly described. Our group has characterized such activation identifying a broad panel of proteins (cellular adhesion molecules, CAMs; e.g., LFA-1, ALCAM, and E-selectin) that are highly upregulated in tumor-associated microglia together with other brain cell types (e.g., endothelial cells and astrocytes) within the TME (Soto et al., 2014). However, it is clear that the macrophage population within the brain TME is not restricted to tissue-resident macrophages (microglia), but also includes recruited bone marrow-derived macrophages (BMDMs) (Bowman et al., 2016). Microglia develop from embryonic yolk sac progenitor cells (Ginhoux et al., 2010) and persist in the CNS into adulthood (Ajami et al., 2007). By contrast, in response to homeostasis disturbance, circulating monocytes are recruited to the brain parenchyma and give rise to BMDMs (Shi and Pamer, 2011). Whether microglia and BMDMs have distinct functions in the brain tumor microenvironment has been controversial, and is a topic of active investigation. Importantly, microglial activation and presence is not just relevant in brain metastasis (Sevenich et al., 2014). For instance, in glioblastoma, the most aggressive form of solid cancer in the CNS, microglia and BMDMs comprise up to 30–50% of the total tumor mass (Badie and Schartner, 2000; Pyonteck et al., 2013), suggesting a critical role for these cell types during tumor progression within the CNS more generally.

Differentiating between microglia and BMDMs is not straightforward. Microglia and infiltrated macrophages share similar morphology, partly imposed by the local microenvironment within the CNS. Differential CD45 expression has been suggested to distinguish between microglia (CD45low) and BMDMs (CD45high) in murine models, however, this does not seem to correlate with human brain tumor studies (Bowman et al., 2016). Recently, genetically engineered mouse models have provided means to distinguish between BMDM and resident microglia (Mizutani et al., 2012). Mizutani and collaborators generated a chimeric mouse where microglial cells were exclusively CX3CR1-GFP+, whilst hematogenous leukocytes were CCR2-RFP+. This model has helped to identify the role of each cell subpopulation during embryogenesis and disease (Bennett et al., 2016). However, these approaches are clearly not of relevance to human investigation (Sevenich, 2018). Very recently, the homeostatic marker Tmem119 has been shown to be enriched on microglia, but not BMDMs, in both human and mouse brain tissue (Bennett et al., 2016). Cx3cr1 (another homeostatic microglial marker) and Siglec-H are also expressed by resting microglia, but neither are expressed by activated microglia or macrophages derived from lung or peritoneum (Sheng et al., 2015). Conversely, CD49D/ITGA4 was identified as a marker for BMDMs (Gautier et al., 2012). Importantly, novel proteomic studies have indicated the influence that the tumor microenvironment may exert on microglia-specific biomarkers, altering the phenotype of the analyzed immune cells and introducing potential confounds to data interpretation (Bowman et al., 2016). Thus, our understanding of how BMDM and microglia, respectively, contribute to metastasis progression in the brain remains in its infancy. Nevertheless, with these improved tools and greater insight into potential confounds, we are now in a position to examine these questions more fully.

## Ambiguous Response

The innate role of the cells of the immune system is to guard, detect and respond to any insult that may disrupt the regular physiological equilibrium or homeostasis. However, as a result of their ability to take on an anti-inflammatory phenotype, microglia/BMDMs are able to advocate a pro-tumorigenic scenario during cancer and metastasis progression Sica et al., 2006; Hao et al., 2012; Roesch et al., 2018). This phenotype exhibits, (i) increased anti-inflammatory cytokine production, (ii) loss of phagocytic activity, (iii) release of growth factors, (iv) chemo-attractive effect on peripheral monocytes, and (v) inhibition of T-cell proliferation within the tumor microenvironment (Quail and Joyce, 2013).

Immune cells with a pro-inflammatory phenotype have been described as exerting an anti-tumorigenic (M1 or classical activation) effect whilst, in contrast, those with an anti-inflammatory profile (M2 or alternative activation) have shown tumor-supporting activity (Pukrop et al., 2010; Vicetti Miguel et al., 2010). The historical view that microglial cells exist in a balance between two states, pro- (M1) and anti-inflammatory (M2), is now considered too simplistic. Many groups are instead focused on defining context-specific microglial activation and phenotype as a measure of functional diversity. Deep transcriptomic analysis has yielded a plethora of microglial genes (e.g., Lgals3, Trem2, NOS2, COX1, etc.) that encompass the Disease-Associate Microglia (DAM). This characteristic microglial pattern was first reported by Keren-Shaul et al. (2017) and aims to characterize the microglial response during neurodegenerative processes. Further studies (Krasemann et al., 2017; Deczkowska et al., 2018) have completed the inflammatory profile of these DAM cells in a broad panel of central diseases (e.g., Multiple Sclerosis, Alzheimer’s disease, Amyotrophic Lateral Sclerosis, or Parkinson’s disease) including primary brain cancer and metastasis to the CNS (Sevenich, 2018). Therefore, it seems reasonable to accept the existence of a complex microglial behavior during central disorders, including brain metastasis, beyond the simplistic M1-M2 definition (Ransohoff, 2016). However, the molecular pathways underlying the mechanistic switch during breast cancer brain metastasis progression are still unknown.

## Perspectives for Therapies Targeting Microglia in Secondary Brain Cancer

The poor bioavailability in the brain of systemically successful treatments, and the impact of complex and variable environmental factors has reduced the efficacy of novel therapies in brain metastasis. In recent years, the inflammatory microenvironment has shown the potential for exploitation as both a prognostic and therapeutic tool in various malignancies. In the following section, we discuss the potential of microglia modulation as a novel therapeutic strategy in BM.

### Immunotherapy

Harnessing the body’s immune response to tumors has recently shown promise for the treatment of different cancer types. Current studies are focusing on the use of check-point blockade treatments not just to stop primary tumor progression, but also their metastasis to distant organs. The clinical success of specific immune checkpoint blockade, such as PD-1 and CTLA-4 (negative regulators of T-cell adaptive immune function), represent a promising advance in cancer immunotherapy development (Pardoll, 2012). An important function of microglial cells (innate immune response) in the inflammatory microenvironment of BM is T-cell activation via expression of the HLA ABC/major histocompatibility antigen class I. Therefore, microglial activation is mandatory for the induction of a specific immune response including T and B cells (Graeber and Streit, 2010).

However, as previously described, microglia cells are also capable of immune suppressive functions. PD-L1 expression, the ligand of the inhibitory T-cell co-receptor PD1, has been detected on microglial cells (Magnus et al., 2005). Via activation of CTLA4 or PD1, microglial cells can suppress the anti-tumor T-cell response and participate in the generation of an immune suppressive tumor microenvironment. Microglia have also been shown to enhance invasion and colonization of brain tissue by cancer cells by serving both as active transporters and guiding rails (Berghoff et al., 2015). In an innovative study, Lorger’s group described the role of combinative anti-PD-1/anti-CTLA-4 therapy in a mouse model of melanoma brain metastasis. They showed the benefit of combining both immune check point drugs in melanoma brain metastasis treatment aiding CD8+ T cell intratumoral infiltration. Nevertheless, understanding of the behavior of the microglial response during immune-check point blockade therapy is still in its early stages.

Although most immunotherapies have been targeted to the adaptive immune response, recent studies have begun to focus on the innate immune response and macrophages in particular. Studies suggest that microglial cells manifest a pro-inflammatory phenotype during cancer progression, whilst BMDM play a more anti-inflammatory role (Cherry et al., 2014; Andreou et al., 2017; Xing et al., 2018). Most pre-clinical models and early phase clinical trials focus on modulating the inflammatory response driven by tumor associated macrophages, rather than depleting them from the organism (Pyonteck et al., 2013; Quail and Joyce, 2013). One of the most studied targets for such phenotypic modulation is the macrophage colony stimulating factor-1 (CSF1). Several studies using CSF1-R inhibitors have proved their efficacy by inhibiting altenative-M2 markers on BMDM in several primary cancers, although its potential role in BM is still to be elucidated. In the same vein, expression of the mannose receptor on peripheral monocytes has been related to anti-inflammatory properties (Chieppa et al., 2003). Furthermore, we recently demonstrated that selective depletion of this mannose receptor-expressing subtype of microglia and BMDMs by intracerebral injection of mannosylated clodronate liposomes in a mouse model of breast cancer brain metastasis significantly reduced BM burden over a time-course study (Andreou et al., 2017).

These promising results in pre-clinical studies could pave the way for future trials in which the immune response could be modulated into a more anti-tumorigenic state. However, we need to be cautious and consider the potential side effects that these drugs may provoke in healthy organs, as a consequence of the overstimulation of the immune system. Nevertheless, the substantial potential gains are likely to outweigh the possible negative side-effects, as has proven the case with both chemotherapies and current immunotherapies.

### Combinative Approaches

Despite the slight improvement in patient life expectancy, the survival for BM patients is still measured just in months, reflecting, in large part, the complex nature of BM disease. Stereotactic radiotherapy and surgery are keystones for the management of many different subtypes of metastatic lesions in the CNS, but still yield relatively little benefit. However, combination of gold-standard therapies such as ionizing radiation with novel anti-tumorigenic drugs may lead the way to achieve better clinical results. For example, anti-CSF1-R therapies in combination with IR or anti-PD-1 are currently ongoing in primary brain tumors (Butowski et al., 2016).

**FIGURE 1:**
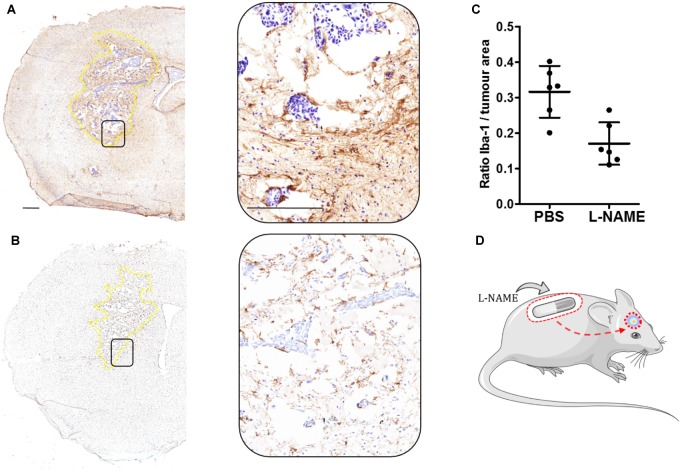
**(A)** Immunohistochemical image of one brain section from the left striatum of a female SCID mouse injected intracranially with 5×10^3^ MDA231Br-GFP cells (Day 21). Microglia activation is stained brown (Iba-1, 1:300: Abcam, United Kingdom). This control mouse was treated with PBS via implanted osmotic minipump throughout the entire time-course study. Inset showing the selected area magnified (20×). Tumor area circumscribed in dotted yellow line. **(B)** As for **(A)** from a mouse treated with L-NAME (30 mg/kg) via osmotic minipump. **(C)** Graph showing the ratio of microglial activation contained within the left striatum as a ratio of tumor area. Quantitation of microglia activation in animals treated with L-NAME (*n* = 6), showed a significant (unpaired Student *t*-test, *p* = 0.003. GraphPad Prism, United States) reduction compared to PBS-treated control (*n* = 6). Iba-1 expression was measured using an Aperio ImageScope^®^ (Leica, Germany) algorithm, in which positive stained (brown) pixels where measured within the left striatum of each animal (*n* = 6 per group). Scale bar = 300 μm. **(D)** Schematic of the experimental model.

Our group has described several cellular adhesion molecules specifically upregulated within the tumor-associated microglial population (Soto et al., 2014, 2016), and inhibition of tumorigenic molecules such as lymphocyte function-associated antigen 1 (LFA-1) and activated leukocyte cellular adhesion molecule (ALCAM) have been shown to significantly reduce breast cancer BM. Additionally, the local inflammatory response to tumor cells, in which microglia play a pivotal role, is regulated by a plethora of pro- and anti-inflammatory cytokines. Moreover, we have shown that activation of enzymes such as nitric oxide synthase (NOS) and cyclooxygenase-2 (COX-2) within the TME is key to tumor progression, and that this is driven by tumor-host cell interactions through LFA-1 signaling. Recent findings from our group have shown that selective inhibition of NOS, by L-NAME administered via osmotic mini-pump, reduced the microglial response in a breast cancer brain metastasis xenograft model (Figure 1).

## Brain Metastasis Treatment Challenges

Advanced treatments for brain metastasis patients consist of a combination of surgery, radiation and the administration of systemic chemotherapeutic drugs. However, these strategies entail a few limitations depending on location, total tumor mass, radio-resistant/sensitive histology, etc. Advances in systemically administered therapies have focused on overcoming these limitations, but the outcome has varied between no effect on metastatic burden, undesirable cognitive and physical side effects, and short transient response on tumor growth arrest with similarly unsuccessful results long-term (Owonikoko et al., 2014). Part of the poor improvement afforded by new targeted therapies reflects restrictions on brain uptake owing to the BBB and the tumor-brain barrier (TBB) (Gril et al., 2018). Consequently, effort is now directed at designing novel strategies to either selectively permeabilise the BBB and TBB at the metastatic *foci* (Connell et al., 2013) or to enhance the ability of systemic therapies to cross the BBB/TBB (Lockman et al., 2010; Gril et al., 2018).

At the same time, a lack of biological understanding of the long-term impact of newly designed drugs on different cell populations within the tumor microenvironment, including microglia, also contributes to the scarce improvement in BM patient outcome. Moreover, whilst the role of T cells, microglia and BMDM in brain metastasis progression has received some attention, the contributions of other immune cells, such as neutrophils, myeloid-derived suppressor cells and dendritic cells, have yet to be considered in any detail.

## Future Directions

Irrespective of the efficacy of novel targets in brain metastasis, it seems highly unlikely that single therapies will overcome this stage of cancer progression. Rather, combinations of gold-standard therapies such as radiotherapy with molecularly targeted brain-specific treatments against key components of the TME may afford increased life expectancy in patients suffering metastatic spread to the brain.

Understanding the impact of immune check point blockade not just on T cell biology, but also on microglial behavior enable further enhancement of the global immune response and overcome the macrophage/microglia-induced tumor supportive scenario observed in BM. At the same time, transcriptomic sequencing of tumor-associated microglia during the course of brain metastasis may improve our understanding of the pathways that ultimately lead to the tumor supportive scenario.

Current clinical trials in neurodegenerative disease aim to reduce the local inflammatory response targeting typical pro-inflammatory microglia biomarkers (Subramaniam and Federoff, 2017). In a brain metastasis context, the reverse is a key goal and future directions should include strategies in which the anti-inflammatory response of microglia is suppressed and their cytotoxic (pro-inflammatory) response promoted.

## Ethics Statement

All animal procedures were performed under the University of Oxford guidelines and approved by the UK Home Office.

## Author Contributions

MS and NS participated in the bibliography search and the design, correction, and completion of this article.

## Conflict of Interest Statement

The authors declare that the research was conducted in the absence of any commercial or financial relationships that could be construed as a potential conflict of interest. The handling Editor declared a shared affiliation, though no other collaboration, with one of the authors MS at the time of review.
